# Alcohol-Related Emergency Department Visits of Young Adults in a University City in the Netherlands

**DOI:** 10.1016/j.acepjo.2026.100519

**Published:** 2026-10-07

**Authors:** Thirza Arentje Helena Noordermeer, Louise Elisabeth Maria Pigeaud, Loes de Veld, Nico van der Lely

**Affiliations:** 1 Department of Pediatrics, Reinier de Graaf Hospital, Delft, The Netherlands; 2 Erasmus School of Health Policy & Management, Erasmus University Rotterdam, Rotterdam, The Netherlands; 3 Faculty of Medicine and Health Science, University Antwerp, Antwerp, Belgium

**Keywords:** acute alcohol intoxication, young adults, emergency department, alcohol-related injuries, students

## Abstract

**Objectives:**

This study aimed to examine the incidence and characteristics of alcohol-related emergency department (ED) visits in young adults compared with older age groups.

**Methods:**

A retrospective electronic health record study of patients aged ≥18 years with an alcohol-related ED visit, including acute alcohol intoxication (AAI) and alcohol-related injuries, was conducted at Reinier de Graaf Gasthuis (RdGG) from January 1, 2022 to January 1, 2024. Young adults (18 to 24 years) were compared with older age groups (25 to 39, 40 to 54, 55 to 69, >70 years) regarding visit type, blood alcohol concentration (BAC), diagnostic tests, and hospital admission. Within young adults, student status and peak visit times were analyzed.

**Results:**

Of 49,226 ED visits, 888 (1.8%) were alcohol-related, including 192 (21.6%) young adults, of whom 40% were students. Men were more frequently admitted with alcohol-related injuries (36.4% vs 25.4%), women for AAI (67.8% vs 47.3%, *P* < .001). Alcohol-related visits were highest among young adults (4.3% vs. 0.7-2.8%, *P* < .001). AAI was significantly more prevalent in the 18 to 24 group than in older groups. Most injuries in young adults involved head or neck trauma (84%). Students had higher mean BACs than nonstudents (2.34 vs 1.84 g/L, *P* = .019).

**Conclusion:**

Young adults account for the highest incidence of alcohol-related ED visits. Students exhibit higher BACs than nonstudents. These findings underscore the critical need to reduce alcohol-related harm and promote safe drinking habits among young adults especially during specific periods.

**The Bottom Line**

Alcohol-related to visits represent a substantial burden on emergency care. We reviewed all adult alcohol-related ED visits over a 2-year period to examine the incidence and characteristics of alcohol-related ED visits in young adults compared with older age groups. Of 49,226 ED visits, 888 (1.8%) were alcohol-related. Young adults aged 18 to 24 years had the highest proportion of alcohol-related visits (4.3%), compared with 0.7% to 2.8% in older age groups. Students had higher mean BACs than nonstudents (2.34 vs 1.84 g/L). These findings suggest that prevention efforts should focus on young adults, particularly students, to reduce alcohol-related harm.

## 1 Introduction

### 1.1 Background

Alcohol consumption has a substantial impact on emergency departments (EDs). Globally, alcohol consumption is frequently associated with ED visits due to acute alcohol intoxication (AAI) and alcohol-related injuries, including road traffic accidents, falls, and drownings.^1^ In the young adults group, alcohol-related incidents, are a primary cause of ambulance call-outs.^2^, ^3^, ^4^ Alcohol consumption accounts for more ED visits than all other illicit drugs (amphetamines, opioids, cannabis, and cocaine) combined.^5^

Previous studies have demonstrated the harmful effects of alcohol. Alcohol use can negatively affects young adults’ physical health, mental well-being, academic performance and social lives.^6^, ^7^, ^8^ Studies presented higher levels of alcohol consumption in students compared with their nonstudent peers.^9^, ^10^, ^11^, ^12^, ^13^ This is often attributed to the social environment of college life, including parties, social gatherings, student associations, and a culture of binge drinking. Within the student population, several risk factors have been identified as contributing for higher alcohol use, including living independently,^14^^,^^15^ social and peer pressure,^16^ stress,^17^, ^18^, ^19^ and membership in student associations,^20^ compared with nonstudents.

### 1.2 Importance

Although various studies have examined the effects of alcohol on young adults, detailed characterization of alcohol-related ED visits in this group, such as BAC, use of diagnostic tests, type of ED visit, and proportion of ED visits, compared with older age groups (>24 years) remains limited. In addition, research focusing specifically on students within the young adult population in the Netherlands is scarce. This study aims to provide insight into this specific group and inform the development of targeted prevention strategies.

### 1.3 Goals of This Investigation

The aim of this study was to ascertain the characteristics and incidence of alcohol-related ED visits in a major general hospital in the Netherlands, located in a city with a high student population. In addition, we aimed to compare young adults (aged 18 to 24 years) with older age groups (>24 years) in terms of alcohol-related ED visit type, blood alcohol concentration (BAC), use of diagnostic tests, and hospital admission rates. Finally, within the young adult group, we analyzed student status and peak times in alcohol-related ED visits.

## 2 Methods

### 2.1 Study Design and Setting

A retrospective electronic health record study (EHR) was performed at the Reinier de Graaf Gasthuis (RdGG). The EHRs were extracted using a search strategy implemented with the screening software CTcue.^21^ The search terms were as follows: “alcohol intoxication,” “ethanol,” “C2H5OH,” “mixed intoxication,” “autointoxication,” BAC ≥ 0.0 and/or Diagnostic and Treatment Combination (DBC)-codes “alcohol intoxication” or “auto-intoxication.” The DBC code system was developed within the Dutch health care system to document diagnoses and treatments, thereby enabling an overview of the associated costs.^22^

### 2.2 Selection of Participants

The study population consisted of patients aged 18 years and older who visited the ED at RdGG, a major general hospital located in the Netherlands, due to an alcohol-related cause between January 1, 2022 and January 1, 2024. Inclusion criteria compromised patients aged ≥ 18 years with an alcohol-related ED visit, including AAI, alcohol-related injury or a combination, during this period. Patients were included if they fulfilled at least 2 of the following 3 criteria: 1) the presence of predefined search terms related to alcohol intoxication, 2) a documented BAC or 3) a relevant DBC code. AAI was defined as a BAC ≥ 0.5 g/L or a BAC <0.5 g/L accompanied by a clinical diagnosis of alcohol intoxication based on clinical signs or symptoms and documented patient-reported alcohol consumption levels consistent with binge drinking (≥4 standard drinks for women and ≥5 standard drinks for men), as recorded in the EHR. Cases with uncertain AAI classification were independently reviewed by 2 authors (LV an NL) to reach consensus and minimize the risk of misclassification. Patients were classified as students if their student status could be clearly established from the available documentation in the EHRs, including occupation information and incident descriptions. Exclusion criteria included patients with an alcohol-related ED visit caused by secondary conditions associated with alcohol consumption, such as withdrawal syndrome, Wernicke–Korsakoff syndrome, alcoholic gastritis, alcoholic hepatitis or alcoholic pancreatitis.

### 2.3 Measurements

The EHRS derived from the search with the screening software CTcue, included ED reports from physicians, nurses, ambulance personnel, general practitioners, and other medical disciplines. The first and second authors manually reviewed all EHRs. In cases of uncertainty regarding inclusion, both authors reviewed the cases together.

### 2.4 Outcomes

The following data were collected from the EHRs: age (years), sex, type of alcohol-related ED visit, BAC (g/L), drug use, additional investigation (radiological tests, laboratory tests, and electrocardiograms [ECGs]), hospital admission, and relevant medical specialist.

All alcohol-related ED visits were considered separate cases. Patients were stratified into the following age groups: 18 to 24, 25 to 39, 40 to 54, 55 to 69, and >70 years. Characteristics compared across age groups include the type of alcohol-related ED visit (AAI, alcohol-related injury or a combination), BAC, use of diagnostic tests, and hospital admission rates. Within the 18 to 24 years age group, student status and peak times of alcohol-related ED visits were analyzed. Student status was characterized based on information from the EHRs, such as drinking location (student house or student association) or type of event (hazing, introduction period, and student party). The data were anonymized coded and stored in SPSS.

### 2.5 Data Analyses

Data were analyzed with the statistical package SPSS (version 25.0 for Windows, 2017; Armonk, NY). Nominal variables were analyzed with chi-square tests. Continuous variables were analyzed with the independent t-test and analysis of variance (ANOVA). Ordinal variables were analyzed with the Mann–Whitney test. Nominal and ordinal variables were described using frequency tables, mode and median. Normally distributed continuous variables were described in means, standard deviations and the 95% confidence intervals. The incidence of alcohol-related ED presentations was calculated as a proportion of the total number of ED visits. Post hoc analyses involved pairwise comparisons using multiple z-tests with Bonferroni correction. A *P* value *P* < 0.05 was considered significant.

## 3 Results

### 3.1 Characteristics of Study Subjects

During the study period, 49,226 patients visited the ED. Of these patients, 1189 were identified with a potential alcohol-related ED visit. Due to misclassification or incomplete data, 231 patients were excluded. Misclassified cases included for instance mentions of AAI in the medical history without relevance to the current ED visit, drug-related intoxications without alcohol involvement or the use of the search term “drinking” in case of kidney problems, urine infections and dehydration. Following a qualitative review of the EHRs of the remaining 958 patients, 70 additional patients were excluded due to secondary alcohol-related conditions, including alcohol withdrawal, Wernicke–Korsakoff syndrome, alcohol-induced hepatitis, pancreatitis or gastritis and alcoholic liver cirrhosis.

### 3.2 Main Results

In total, 888 patients had alcohol-related ED visits, representing 1.8% of all ED presentations in the RdGG during the study period. The study population consisted of 577 (65%) men and 311 (35%) women, with a mean age of 45 years. Of the 888 patients, 192 (21.6%) were in the 18 to 24-year old group, of whom 76 (40%) were identified as students.

#### 3.2.1 Differences between age groups

Patients with alcohol-related ED visits were categorized in the following different age groups: 18 to 24, 25 to 39, 40 to 54, 55 to 69, and >70 years (Table 1). Young adults aged 18 to 24 years had a lower absolute number (n = 192, 21.6%) of total ED visits (n = 888), but a significant higher incidence (4.3%) of alcohol-related ED visits, *P* < .001 compared with older age groups. More than half (57.8%) of the alcohol-related ED visits in the 18 to 24 age group were attributable to AAI. Post hoc analysis using pairwise comparisons with Bonferroni correction demonstrated that. AAI was significantly more prevalent in the 18 to 24 group compared with the older groups (55 to 69 and >70). In contrast, alcohol-related injuries were more prevalent in the older age groups (55 to 69 and >70) compared with the 25–39 age group and the 40–54 age group ( 41.6 to 53.0% vs 22.6 to 23.9%, *P* < .001). No differences in sex distribution were observed across age categories. There was a significant association between sex and type of visit in the overall population (*P* < .001), with males more frequently presenting with alcohol-related injuries (36.4% vs 25.4%) and females with AAI (67.8% vs 47.3%). A combined presentation was more common among men than women (16.3% vs 6.8%, *P* < 0.001). Post hoc analysis using pairwise comparisons with Bonferroni correction showed that men were more frequently admitted with alcohol-related injuries or a combination of such injuries whereas women were more commonly admitted due to AAI (Table 1).

**Table 1:** Incidence of alcohol-related emergency department visits stratified by age 2022-2024.

	Age categories					*P* value
	18-24 years	25-39 years	40-54 years	55-69 years	≥70 years	
N alcohol-related ED visits	192	177	226	178	115	<.001
N ED visits	4485	7901	7961	11,149	17,730	
*%* Alcohol-related, n (%)	4.3	2.2	2.8	1.6	0.7	
Sex, n (%)						
Male	115 (59.9)	121 (68.4)	148 (65.5)	122 (68.5)	71 (61.7)	
Female	77 (40.1)	56 (31.6)	78 (34.5)	56 (31.5)	44 (38.3)	.329
Type of ED visit, n (%)						
Alcohol intoxication	111 (57.8)^a^^,^^b^	121 (68.4)^a^	142 (62.8)^a^	74 (41.6)^b^	36 (31.3)^b^	
Alcohol-related injury	60 (31.3)^a^^,^^b^	40 (22.6)^b^	54 (23.9)^b^	74 (41.6)^a^^,^^c^	61 (53)^c^	
Combination	21 (10.9)^a^^,^^b^	16 (9)^a^	30 (13.3)^a^	30 (16.9)^a^^,^^b^	18 (15.7)^a^	<.001

ED, emergency department; N, number.

Chi-square tests with Bonferonni adjusted post hoc tests.

a-c Within a row, means without common superscript differ (*P* < .05).

#### 3.2.2 Young adults versus >24 years

The characteristics of alcohol-related visits among the 18 to 24 age group compared with the >24-year-old group were presented in Table 2. The BAC was significantly lower in young adults (M = 2.09 g/L, SD = 0.87) than in the >24 years group (M = 2.32 g/L, SD = 1.14), t(330.69) = −2.738, *P* = .007. In addition, the proportion of patients transported by ambulance was significantly lower in young adults compared with the >24-year-old group (77.4% vs 85.4%, *P* = .008). In the young adult group, 167 (87%) laboratory tests, 58 (30.5%) ECGs and 76 (40%) radiological tests were conducted. Urine toxicology screening was rarely performed (11%). The majority of the group with alcohol-related injury had head or neck trauma (80.4% to 84%). The admission rate in the young adult group was 43.9%.

**Table 2:** Intoxication characteristics between 18-24-years and >24 years.

	Age categories		*P* value
	18-24 years	>24 years	
N alcohol-related ED visits	192	696	
BAC (g/L)	2.09 ± 0.88	2.32 ± 1.14	.007^d^
Ambulance, n (%)	147 (77.4)	589 (85.4)	.008
Hospital admission	83 (43.9)	371 (53.6)	.018
ECG	58 (30.5)	303 (43.8)	.001
Radiology tests	76 (40)	463 (52.5)	<.001
Laboratory test, n (%)			
Performed	167 (87)	638 (91.7)	
Not performed	25 (13)	58 (8.3)	.048
Urine drug screening, n (%)			
Positive	7 (3.7)	29 (4.2)	
Negative	14 (7.3)	58 (8.3)	
Not performed	170 (89)	608 (87.5)	.850
Alcohol-related injuries, n (%)			
Head/neck	68 (84)^a^	258 (80.4)^a^	
Torso/spine	2 (2.5)^a^	14 (4.4)^a^	
Shoulder/arm/hand	5 (6.2)^a^	17 (5.3)^a^	
Hip/leg/foot	3 (3.7)^a^	17 (5.3)^a^	
Other	3 (3.7)^a^	15 (4.7)^a^	.871

BAC, blood alcohol concentration; ED, emergency department; N, number.

a-c Within a row, means without common superscript differ (*P* < .05)

d Independent-samples t-test and chi-square tests with Bonferonni adjusted post hoc tests.

#### 3.2.3 Student status

Characteristics of alcohol-related ED visits according to student status among the total study population were presented in Table 3. A total of 76 (8.6%) patients were identified as students, 698 (87.8%) as nonstudents and the status was unknown for 112 (12.6%). There was no difference in the distribution of causes for alcohol-related ED visits between students and nonstudents. Students had a significantly higher mean BAC than the nonstudents (2.34 vs 1.84 g/L, *P* = .019) (Table 3).

**Table 3:** Relation between student status and type of alcohol-related ED visit among all patients.

	Student status			*P* value
	Student	No student	Unknown	
N alcohol-related ED visits, n (%)	76 (8.6)	698 (87.8)	112 (12.6)	
BAC (g/L)	2.34 ± 0.89	1.84 ± 0.82	1.98 ± 0.85	.019^a^
Type of ED visit, n (%)				
Alcohol intoxication	42 (55.3)	379 (54.3)	61 (54.5)	
Alcohol-related injury	24 (31.6)	225 (32.2)	40 (35.7)	
Combination	10 (13.2)	94 (13.5)	11 (9.8)	.846

BAC, blood alcohol concentration; ED, emergency department; N, number.

a One-way ANOVA-test and chi-square test.

Exploratory inspection of the monthly distribution suggested relatively higher numbers of alcohol-related visits ED visits among students in February–March, August and December. These observations are descriptive only, as the sample size was too small to permit a chi-squared test or trend analysis.

## 4 Limitations

Several methodological considerations should be taken into account when interpreting the findings of this study.

First, alcohol-related emergency department visits were identified using predefined electronic search terms and diagnostic codes extracted from the EHR with CTcue. Consequently, visits in which alcohol use was not documented or coded may not have been captured, resulting in an underestimation of the true burden of alcohol-related ED presentations. In addition, the absence of data from general practitioner out-of-hours services may have further underestimated the overall burden of alcohol-related acute health care utilization.

Second, the reported incidence was calculated as the proportion of alcohol-related ED visits among all ED visits within each age group. Because overall ED utilization differs substantially between age groups, these estimates should be interpreted with caution and should not be considered equivalent to population-based incidence rates or age-specific risks of alcohol-related ED presentation. As this observational study was conducted in a university city with a specific demographic profile, the relative overrepresentation of young male adults may limit the generalizability of our findings, particularly regarding analyses of sex distribution and BAC levels, to other cities in the Netherland.

Third, we excluded secondary alcohol-related conditions, including alcohol withdrawal, Wernicke–Korsakoff syndrome, alcoholic hepatitis, pancreatitis, and cirrhosis. As these conditions are more prevalent among older adults, their exclusion may have led to an underestimation of the absolute number of alcohol-related ED visits in this age group. However, these conditions are frequently associated with recurrent ED presentations, and their inclusion could have disproportionately increased the number of visits among older patients, potentially biasing comparisons between age groups. Furthermore, alcohol-related injuries were not always explicitly documented in the EHR, which may have resulted in additional under ascertainment of alcohol-related presentations.

Finally, the subgroup of patients identified as students was relatively small (n = 76), and student status was unknown for 12.6% of eligible patients. Student status was determined retrospectively from narrative clinical documentation rather than a standardized EHR variable, introducing the possibility of misclassification bias. To minimize this risk, a conservative classification strategy was applied, whereby patients were classified as students only when this status was explicitly documented in the medical record. The retrospective study design inevitably resulted in missing or incomplete data because student status and other relevant variables were not consistently recorded in routine clinical practice. Nevertheless, the limited sample size precluded formal analyses of temporal trends among students. Although descriptive analyses suggested seasonal peaks in alcohol-related ED visits during February–March, August, and December, these observations should be interpreted cautiously.

## 4 Discussion

### 4.1 Main Findings

The aim of this study was to ascertain the characteristics and incidence of alcohol-related ED visits in the young adult group compared with older age groups, focusing on type of alcohol-related ED visit, BAC, use of diagnostic tests, and hospital admission rates. Within the young adult group, student status and peak times of alcohol-related ED visits were analyzed.

This study demonstrated a significantly higher incidence of alcohol-related ED visits in the young adult group compared with the age categories of patients aged 25 years and older. AAI was significantly more prevalent in the young adult group, whereas alcohol-related injuries predominated in the older groups (55 to 69 and >70). Most alcohol-related injuries involved head or neck trauma. Furthermore, students had a significantly higher BAC compared with nonstudents. The results suggest peaks in alcohol-related ED visits among students during February–March, August, and December.

The significantly higher incidence of alcohol-related ED visits among young adults is consistent with findings from a previous study. A recent Australian study among 43 hospitals demonstrated a similar peak-incidence of alcohol-related ED visits in the 18–24 age group.^23^ In contrast, data from the American Nationwide Public Health Surveillance System present a peak in the age group of 26 to 44 years.^6^

When the type of alcohol-related visits was examined in more detail, more than half of the alcohol-related ED visits in the 18 to 24 age group were attributable to AAI. This finding is consistent with Dutch national data from 2020, in which most patients presenting with AAI were in the underage group (12 to 17), followed by the 18 to 24 age group.^2^ In contrast to the predominance of AAI in the younger age groups, alcohol-related injuries were the predominant type of alcohol-related ED visits in the older age groups (>55 years). In both age groups, the majority of the patients with alcohol-related injuries had head or neck trauma (>80%). The predominance of AAI in the younger age groups coincides with the peak of heavy episodic drinking or binge drinking, defined as consumption of ≥5 alcoholic drinks for men and ≥4 alcoholic drinks for women on one occasion.^24^, ^25^, ^26^, ^27^, ^28^

Regarding sex distribution, this study reveals a significantly higher proportion of alcohol-related ED visits by men (65%) compared with women (35%). Globally, alcohol consumption is more prevalent among men than women, although the extent of this difference varies across cultures and economic contexts.^1^ Most alcohol-related indicators present higher values among males than females. Consequently, alcohol-related ED visits are more common among men than women.^2^ With a male-to-female ratio of 1.9:1, the predominance of males in alcohol-related ED visits in Delft is higher than might be expected from a high-income country.^29^ This may partly be explained by demographic characteristics of the local population, particularly the male predominance in the 20 to 30 year age group, which is associated with the relatively high proportion of male students attending the Technical University in Delft. Similar demographic patterns have been observed in other Dutch cities with technical universities, such as Eindhoven and Twente.^29^

A notable strength of this study is its reliable presentation of the study population of alcohol-related ED visits in the Netherlands. The percentage of alcohol-related ED visits observed in this study is in line with the findings from a recent observational study investigating the impact of the COVID-19 pandemic on alcohol-related ED visits.^30^ Additionally, a national Dutch study using data from a representative sample of ED visits in 2020, reported similar percentages of AAI and alcohol-related injuries in the 18 to 24 age group.^31^

In this study, BAC was assessed in 90.5% of the patients with an alcohol-related ED visit. Accurate assessment of the degree of AAI may be an essential step in the treatment of patients in ED setting. An international study demonstrated that the level of BAC does not correlate with the clinical signs of patients with AAI, particularly in individuals with chronic alcohol consumption.^24^^,^^32^ The accuracy of clinicians in estimating BAC based on their clinical experience is limited.^33^ BAC was significantly lower in the 18 to 24 group compared with the older group, despite the higher incidence of AAI in this group. Similarly, findings have been reported in 2 international studies.^25^^,^^34^ A possible explanation for this difference might be increased tolerance to alcohol in the older group resulting in a higher BAC.^35^

Among the total study population, it was observed that students had a significantly higher BAC compared with nonstudents. Student status was determined retrospectively from clinical documentation rather than from a standardized field. This approach may influence the comparison between students and nonstudents, and should therefore be considered when interpreting these findings. A hypothesis for this result could be information bias, as patients with a higher BAC may have been more likely to be questioned about social context and student context. However, another possible explanation could be that students, due to their social environment, including student associations and student houses, engage in more frequent and higher levels of alcohol consumption than their nonstudent peers. Data from questionnaires conducted at various educational institutions in the Netherlands support this hypothesis.^13^ Similar findings have also been reported internationally.^33^^,^^36^^,^^37^

An exploratory assessment of the monthly distribution of alcohol-related ED visits among students suggested relatively higher numbers of visits during February–March, August, and December. However, the sample size was too small to perform a formal trend analysis. Although no conclusions regarding seasonal patterns van be drawn, the exploratory monthly distribution was discussed with representatives from the alcohol covenant in Delft. The suggested that the apparent increase in August may be related with the introduction period for new students in Delft, which typically includes a series of events associated with increased alcohol consumption, such as pub crawls and a beer cantus.^20^^,^^38^ The apparent increase in December may be related to Christmas parties and New Year’s Eve celebrations in the 18–24 age group. Lower incidences observed during other months may reflect examination periods, whereas the apparent increase in February–March may correspond to the postexamination period, during which increased alcohol consumption is common. These possible explanations are speculative and require confirmation in larger studies with sufficient power to formally assess seasonal trends.

The incidence of alcohol-related ED visits is the highest in the young adult group. In this group, the students had a significantly higher BAC. The sample size was too small to conduct a time trend analysis test. Further research should aim to include a larger student population, for example by routinely recording this information at the EDs nationwide in cities with high student populations. In addition, the retrospective study design inevitably resulted in missing or incomplete data because student status and other relevant variables were not consistently recorded in routine clinical practice.

In summary, this study demonstrated a substantially higher incidence of alcohol-related ED visits among young adults than among other age groups. Within this population, students presented with significantly higher blood alcohol concentrations than nonstudents. These findings highlight young adults, particularly students, as an important target group for preventive interventions aimed at reducing excessive alcohol consumption and its associated health risks. Prevention efforts could be implemented through municipalities, student associations, health care institutes, and university orientation programs. Furthermore, an alcohol-related ED visit may provide a valuable opportunity to identify individuals at risk of hazardous alcohol use, initiate brief interventions, and facilitate referral to specialized addiction services when appropriate. Future qualitative research is warranted to improve understanding of the social and behavioral factors underlying excessive alcohol use among young adults, thereby supporting the development of more effective and targeted prevention strategies.

## Author Contributions

TN, LV, and NL conceived and designed the study. TN and LV collected the data for this study and performed the statistical analyses. TN drafted the manuscript, and all authors critically revised and approved the final version. TN takes responsibility for the paper as a whole. The authors declare no conflicts of interest.

## Ethics declaration

The authors have NOT obtained informed consent from participants or their legal representatives. The Medical Research Ethics Committee Leiden Den Haag Delft determined that the study did not fall within the scope of the Dutch Medical Research Involving Human Subjects Act (WMO) and was therefore exempt from review (reference number N23.053). The Data Protection Officer and the Research Board of Reinier de Graaf Hospital approved a waiver of informed consent for this retrospective medical record study, based on the use of the CTcue software application, which limits researchers’ access to directly identifiable patient information.

This study was performed in compliance with relevant laws, regulatory frameworks and guidelines where the research took place. The Medical Research Ethics Committee Leiden Den Haag Delft. Reference number N23.053. granted an exemption. The authors provided the following explanation: Ethical approval was not required because the study did not fall within the scope of the Dutch Medical Research Involving Human Subjects Act (WMO). The Medical Research Ethics Committee Leiden Den Haag Delft reviewed the study protocol and determined that the WMO did not apply. The study was therefore exempt from review (reference number N23.053).

## Funding and Support

By *JACEP Open* policy, all authors are required to disclose any and all commercial, financial, and other relationships in any way related to the subject of this article as per ICMJE conflict of interest guidelines (see www.icmje.org). The authors have stated that no such relationships exist.

## Data Sharing Statement

Data will be available upon reasonable request to the corresponding author.

## Conflicts of Interests

All authors have affirmed they have no conflicts of interest to declare.
